# Tolerating tigers: Gaining local and spiritual perspectives on human-tiger interactions in Sumatra through rural community interviews

**DOI:** 10.1371/journal.pone.0201447

**Published:** 2018-11-14

**Authors:** Jeanne E. McKay, Freya A. V. St. John, Abishek Harihar, Deborah Martyr, Nigel Leader-Williams, Betty Milliyanawati, Ika Agustin, Yulian Anggriawan, Karlina, Erlinda Kartika, Fachruddin Mangunjaya, Matthew J. Struebig, Matthew Linkie

**Affiliations:** 1 Durrell Institute of Conservation and Ecology, University of Kent, Canterbury, United Kingdom; 2 Panthera, New York, United States of America; 3 Nature Conservation Foundation, Mysore, India; 4 Fauna & Flora International, Indonesia Programme, Jakarta, Indonesia; 5 Department of Geography, Cambridge University, Cambridge, United Kingdom; 6 Indonesian Ministry of Environment and Forestry, Padang, West Sumatra, Indonesia; 7 Faculty of Biology, Universitas Nasional Jakarta, Indonesia; 8 Wildlife Conservation Society, Indonesia Program, Bogor, Indonesia; University of Tasmania, AUSTRALIA

## Abstract

Religious beliefs and spiritual connections to biodiversity have the potential to reduce animosity towards wildlife that might otherwise present a real or perceived threat to local people. Understanding this social dynamic can therefore be important for formulating locally-appropriate species-specific conservation strategies. Using semi-structured interviews which incorporated human-tiger conflict scenarios, we investigated how beliefs towards tigers varied between ethnic groups living around a large protected area that is home to the largest tiger population in Sumatra. We gathered this information to determine the degree to which cultural tolerance may contribute to the survival of the tiger in the Kerinci Seblat landscape, Indonesia. From 154 interviewees, 133 respondents came from three main ethnic groups, Minangkabau, Kerincinese and Melayu. The majority (73.5%) of Minangkabau interviewees cited that their ethnic group had customary laws regarding tigers, as did 52% of Melayu and 44% of Kerincinese. Irrespective of ethnicity, most participants did not perceive there to be a connection between Islam and tigers. All participants acknowledged the existence of zoological tigers and two groups (Minangkabau and Kerincinese) held a strong common belief that different types of spirit tigers also existed. From presenting different human-tiger conflict scenarios, with varying levels of severity towards livestock or human life, an unprovoked tiger attack in the village elicited the most calls for the tiger to be killed. Yet, if a village or family member was killed by a tiger whilst hunting in the forest then most respondents across all ethnic groups said to do nothing. The frequency of this response increased if a tiger killed someone in the village who had committed adultery, reflecting beliefs associated with the role of the tiger as an enforcer of moral rule. Our study highlights the importance of consulting with local communities who live in close proximity to large and potentially dangerous carnivores when developing conflict mitigation strategies, which hitherto has not been the case in Sumatra.

## Introduction

People are likely to come into greater contact with wildlife as rural populations grow. At worst, animals may be perceived to threaten human lives or livelihoods, and they may be killed in retribution [1]. Conflicts increasingly pose threats to wide-ranging species such as large and potentially dangerous carnivores [2]. However, local opinions and behaviour towards carnivores can differ greatly within and across human societies and landscapes. For example, despite a lack of compensation schemes for livestock losses, many people living in the Kruger area, South Africa, do not kill lions in retribution [3] and there is evidence of cultural tolerance enabling higher persistence of multiple carnivore species in India [4]. However, the persecution of some species continues, often to offset economic loss or to reduce perceived fear of encounters, as found with the hunting of wolves in North America [5].

Tigers have undergone abrupt declines across much of their former range as a result of retribution killings [6] and poaching driven largely by demand for body parts [7]. Increased persecution is frequently observed where local tolerance for tigers has declined and this, in turn, presents an opportunity that is exploited by poachers [8]. The fate of the endangered tiger is concerning because it serves as both a flagship species and a cultural icon that is often used to protect biodiversity and their forest ecosystems [9]. Elucidating any factors that contribute to local tolerance should help ensure a future for tigers, while also informing how conservation conflicts may be managed elsewhere [10].

On the Indonesian island of Sumatra, indigenous people coexist with wild tigers, which is a centuries old tradition in many areas [11]. In the two largest tiger landscapes of Aceh and Kerinci Seblat, habitat conversion has been to smallholder farms rather than to large-scale plantations, as a result, forest habitats in these regions are largely bordered by farmland. A Sumatra-wide tiger survey found that these border forest areas still contain many tigers that co-exist against a backdrop of ongoing encounters with people [12]. It is remarkable that so many tigers should persist in Sumatra, despite the unprecedented demand for their body parts and the weak governance that extends across forest landscapes which support the main subpopulations of tiger [13, 14]. The differences in tolerance towards tigers that have been observed in Sumatra may in part be explained by the widespread acceptance of Islam, which prohibits eating wild boar, a principal tiger prey species, and killing of animals that hunt with their claws such as tigers [15]. In Islam, the role of humans as guardians (*Khalifah*) of God’s creations is also prominently mentioned. However, religion alone does not fully explain variations in tolerance between different ethnic groups that are all Muslim [15, 16].

Some of Sumatra’s indigenous farming communities hold ambivalent attitudes towards tigers. However, certain ethnic groups place the tiger within a cosmological framework that makes its behaviour understandable, less feared and therefore less of a threat [17]. For example, the Kerinci people have lost kin to tigers through the centuries, but often ascribe the meaning to ancestral-tiger lore, in which the victim is considered to have transgressed a customary rule, such as committing adultery or unfairly dividing an inheritance [17]. The Minangkabau in West Sumatra believe that the soul of someone who sinned can transfer to a tiger, becoming a protector in the forest to its human family and friends. The existence on Sumatra of ethnic groups holding different personal and sociocultural beliefs, presents a unique opportunity to investigate how these varied beliefs might offset hostility towards large and potentially dangerous carnivores locally, and elsewhere.

In this study, we sought to gain insights into the socio-cultural situation surrounding a large forested landscape where local encounters with tigers occur but do not always result in retaliation, such as the killing or capture of a tiger. More specifically, we aim to explore associations between ethnicity and: (i) customary and religious beliefs associated with tigers; (ii) responses to hypothetical encounters with tigers; and (iii) the different types of tigers that are perceived locally to be in the forest. Such information will elucidate the potential to strengthen tiger conservation across the landscape by better understanding locally held tiger-related beliefs.

## Materials and methods

### Study area

The 13,791 km^2^ Kerinci Seblat National Park has an elongated shape that spans four provinces (Jambi, Bengkulu, West Sumatra and South Sumatra). The evergreen rainforest of the park is abutted by a hard edge of mainly smallholder farmland supporting crops including coffee, cocoa and cinnamon, as well as industrial oil palm plantations in the south. Kerinci Seblat National Park is listed as a natural UNESCO World Heritage Site because of its rich biodiversity, which includes a globally significant population of tigers [18]. Unlike many other parts of Asia where tiger populations in human-dominated landscapes have been extirpated, Kerinci Seblat National Park not only contains a large tiger population, but also one that lives in close proximity to people [12, 19]. The national park operates six law enforcement patrol teams to deter poaching of tigers and their ungulate prey. These teams include members of local communities who are supported by a local informant network operating widely across the landscape to good effect [20, 21].

There are seven main ethnic groups (Kerincinese, Javanese, Minangkabau, Melayu, Rejang, Pekal and Sanat nomadic hunters) living in the Kerinci Seblat landscape. The three main ethnic groups interviewed in our study were the Kerincinese, Minangkabau and the Melayu Found mainly in the Kerinci District, Jambi Province, the Kerinci people live in an enclave surrounded by the national park. They are primarily a community of coffee, cinnamon and rice farmers, and predominantly follow Islam. However, shamanism and ancestral worship endure, as do rules and prohibitions for those wishing to enter the forest [22]. The Minangkabau, who are the largest matrilineal society in the world, are indigenous to the Minangkabau Highlands of West Sumatra Province. They are also predominantly Muslim and the basis of their customary law, known as Minangkabau *adat*, is strongly influenced by the concept that nature should be viewed as a teacher. Their most important natural law is growth, which in nature is nurtured by the sun and the moon and in a human context, by the mother, resulting in its matrilineal system [15]. The Melayu inhabit parts of North Sumatra, Riau, Jambi and South Sumatra provinces and share ancestry with the dominant ethnic group on the Malaysian peninsula. Early Malays were largely animistic with many elements remaining following the introduction of Islam [23].

### Data collection

Data collection was embedded within a larger questionnaire-based study which used information on 228 human-tiger incidents, reported by local people to the Kerinci Seblat National Park tiger response team, to stratify sampling across the landscape according to incident density (high, medium or low) [24]. Data were gathered from a systematic sample of male and female heads-or-households residing in 11 areas surrounding the national park (four high, three medium and four low incident density). The last seven questions in the questionnaire (Table 1) served as a filter triggering an invite for participation in this study which used semi-structured interviews to gain greater insight into people’s relationships with tigers from those identified as having particularly strong tiger-related beliefs. In addition, in order to maximise data collection from individuals perceived to have strong spiritual beliefs towards tigers, or tiger-related stories, snowball sampling, whereby enumerators asked village heads and questionnaire respondents if they knew anyone fulfilling such criteria, was also adopted.

**Table 1:** Filter questions used to identify individuals with strong tiger-related beliefs. Answers to statements (a) to (f) were given on a five-point Likert scale ranging from strongly agree to strongly disagree. A yes/no response was recorded for (g). Respondents strongly agreeing or agreeing to any of the items (a-f), or answering yes to question (g), were invited for interview.

Statement
a) Tigers have souls
b) People and tigers can exchange souls
c) Tigers will come to the village if someone has done something wrong
d) There are were tigers
e) Tigers are our ancestors
f) Tigers protect us when we are in the forest
g) Do you have any more stories about tigers? For example, *[interviewers to list all items where answer was ‘Strongly agree’ or ‘Agree’]*.

The semi-structured interview contained closed-answer (yes/no) and open-ended questions designed to explore the associations between ethnicity and: (i) customary laws and Islamic beliefs associated with tigers; (ii) the existence of tiger shaman; (iii) responses to human-tiger conflict scenarios; and, (iv) different types of tiger perceived to be living in the study area. Socio-demographic data including sex, age, number of years of formal education and of residence in village, and main source of income were also recorded (S1 and S2 Files).

Closed-answer questions were used to investigate the prevalence of customary laws and religious teachings about tigers and whether or not respondents believed in tiger shaman. Where affirmative responses were given, interviewees were asked for further details. For example, with respect to tiger shaman, open-ended questions explored whether interviewees had a story about a tiger shaman, or if they knew of one in their village or elsewhere in Sumatra.

Next, eleven human-tiger conflict scenarios were presented to respondents as open-ended questions. The scenarios ranged from the benign (e.g. a tiger passes through your village), to the more severe (e.g. a tiger kills your livestock) and the severest (e.g. a tiger kills your friend whilst he is out in the forest hunting for non-tiger prey). These questions aimed to gather local perspectives on human-tiger conflicts that occur in the human domain (village) and the tiger domain (forest); and under conditions that would likely be associated with either a zoological tiger (such as livestock depredation), or a spirit tiger (e.g. a village member is killed by a tiger for committing adultery). Six main themes to these open-ended questions were identified and coded as: (i) do nothing; (ii) kill the tiger; (iii) report the incident to the authorities; (iv) relocate the tiger to another forest; (v) permanently remove the tiger from the wild; and, (vi) other.

Finally, given the strong belief systems that have previously been identified from the Kerinci Seblat landscape [16], respondents were asked to describe the different types of tiger that they believed to live in the forests near their village. This included information on: the types of tiger (zoological or spirit) villagers recognise as living in their forest and their physical characteristics; stories and anecdotes concerning incidents involving any type of tiger; tiger folk-lore, including references to its role in the forest, connection with the village and human-tiger associations; and, any different tiger-related perspectives within and between local ethnic groups. These qualitative data were coded in order to estimate the prevalence of different types of tiger and to identify quotes illustrative of typical examples and experiences.

Data were collected between October 2014 and July 2016 after piloting the survey. Interviews were conducted in the Indonesian language with one or two interviewers present. Interviews lasted between two and three hours. No financial incentives were offered to interviewees. However, they were offered snacks and cigarettes during the interview as this is considered a friendly and polite gesture that is culturally appropriate in the study landscape and it helps creates a relaxed atmosphere. To ensure anonymity, we did not record the names or addresses of interviewees. With permission, interviews were recorded using a Dictaphone or transcribed directly.

### Data analysis

Data from open-ended questions were transcribed in full (Microsoft Word) at the soonest opportunity; coding was conducted in Excel. Descriptive statistics of demographic and fixed-answer questions were generated Microsoft Excel. Pearson’s chi-squared test, performed in R version 3.2.0, were used to investigate differences between ethnicity and the prevalence of tiger-specific customary laws and Islamic beliefs, understanding of *Khalifa* and belief in, and presence of tiger shaman.

### Ethics statement

Ethical approval was granted by the School of Anthropology and Conservation Research Ethics Advisory Group, University of Kent. Free prior informed consent was obtained verbally from all participants. The University of Kent partner, FFI, collected the field data for this project. It operates in Indonesia under an institutional memorandum of understanding with the Ministry of Environment and Forestry.

## Results

Semi-structured interviews were administered to 154 respondents from 67 villages; 100 were identified via the filter questions in [24] and 54 through snowball sampling. The majority of interviewees were male (88.3%) and the average age was 54 years (range 18–90 years). Most (45.5%) had completed elementary school as their highest level of education, followed by senior high (22.1%), junior school (20.1%), or another type of formal education (12.3%). Most (78.5%) were born in the village where they were interviewed. The main occupation for respondents was farming (83.7%). All respondents were Muslim and tended to be from one of three ethnic groups, Melayu (*n* = 50, 32.5%), Minangkabau (*n* = 49, 31.8%), Kerincinese (*n* = 34, 22.1%), or other (*n* = 21, 13.6%). ‘Other’ consisted of five groups (Javanese (*n* = 5), Sudanese (*n* = 2), Rejang (*n* = 6), Palembang (*n* = 1) and mixed (*n* = 7).

### Ethnicity and tigers

Focussing on the three main ethnic groups interviewed, most Minangkabau (73.5%) cited that their ethnic group had customary laws towards the tiger (Table 2). A significantly lower proportion of Melayu (52.0%) and Kerincinese (44.1%) reported the existence of tiger-specific customary laws (X^2^ 8.882, *p* = 0.031). Overall, there was little support for the belief that there was a connection between Islam and tigers, although significantly more Minagkabau (22.4%) stated that there was (X^2^ 8.725, *p* = 0.033). There was no significant difference between ethnic groups and their level of understanding of the term *Khalifa*, which defines the role of humans as guardians (*Khalifah*) of God’s creations (X^2^ 2.202, *p* = 0.531). Amongst those who understood the term *Khalifa* (*n* = 78), most reported that there was no connection to tigers; this believe was most prevalent amongst Kerincinese (78.6%), followed by the Minangkabau (68.0%) and Melayu (60.7%).

**Table 2:** The percentage of interviewees from different ethnic groups reporting the existence (Yes) or absence (No) of tiger-specific customary laws and Islamic beliefs together with the prevalence of *khalifa* understanding.

Ethnic Group	Number of respondents	Customary laws regarding tigers (%)		Islamic beliefs regarding tigers (%)		Understand the term *Khalifa* (%)	
		Yes	No	Yes	No	Yes	No
**Minangkabau**	49	73.5	26.5	22.4	77.6	49.0	51.0
**Kerincinese**	34	44.1	55.9	8.8	91.2	58.8	41.2
**Melayu**	50	52.0	48.0	4.0	96.0	44.0	56.0
**Other**	21	66.7	33.3	9.5	90.5	57.1	42.9
**Total**	154	59.1	40.9	11.7	88.3	50.6	49.4

Regardless of ethnicity, most interviewees believed in tiger shaman (61.7%), but few knew of a tiger shaman living in their village (10.4%), or believed there were tiger shamans elsewhere in Sumatra (16.2%) (Table 3). There were no significant differences between ethnic groups and whether they believed in tiger shamans (X^2^ 0.196, *p* = 0.978), or whether they believed there were shamans elsewhere in Sumatra (X^2^ 6.879, *p* = 0.075). However, Kerincinese respondents were significantly more likely to report there being a tiger shaman in their village (X^2^ 17.518, *p* = 0.001). Typical Kerincinese narratives concerning tiger shaman are captured in this quote, *“When I was young there was a tiger shaman in this village*. *He helped our village capture a [zoological] tiger that came to the village*. *He captured the tiger and killed it*, *after it came to a cage he made*. *He captured this tiger because it had killed many livestock in the village*.*”*

**Table 3:** The percentage of interviewees from each ethnic group reporting whether they believe in tiger shaman and whether tiger shaman may be found in their village or elsewhere in Sumatra.

Ethnic group	Number of respondents	Do you believe that there are tiger shamans? (%)		Does you village have a tiger shaman? (%)		Are there tiger shamans elsewhere in Sumatra? (%)	
		Yes	No	Yes	No	Yes	No
Minangkabau	49	61.2	38.8	4.1	95.9	20.4	79.6
Kerincense	34	64.7	35.3	29.4	70.6	17.6	82.4
Melayu	50	60.0	40.0	4.0	96.0	6.0	94.0
Other	21	61.9	38.1	9.5	90.5	28.6	71.4
**Total**	154	61.7	38.3	10.4	89.6	16.2	83.8

### Human-tiger conflict scenarios

Table 4 presents responses to the human-tiger conflict scenarios broken down by ethnicity. For each scenario, one of three responses, which we discuss further, was typically given: (i) Do nothing; (ii) Kill tiger; and (iii) Report to the authorities. A general pattern observed in the data was that for nearly all of the scenarios presented, most respondents, irrespective of ethnic group, suggested doing nothing to the tiger. Where there was no overall consensus, the conflict scenario related to an unexplained loss. For example, if a tiger killed livestock, either belonging to the respondent or a village member, the Minangkabau were more likely to suggest that the tiger be killed (#2 46.7% and #3 47.4%) than an alternative management option, whereas the Kerincinese were least likely to do this (#2 11.5% and #3 9.1%), being more inclined to report the incident to the authorities (#2 38.5%, #3 36.4%); the Melayu were split between the three responses of do nothing, kill the tiger, or report it to the authorities (#2 46.7%, 31.1% 20.0% and #3 43.8%, 28.1%, 28.1%). This type of response pattern between ethnic groups was also found for the two scenarios where a person was killed for an unprovoked attacked (#4 and 5). However, if a village member or a family member was killed by a tiger whilst out hunting in the forest, then the typical response for all ethnic groups was to do nothing (#6 and #7), with the exception of the Kerincinese; 45.5% would report the killing of their brother by a tiger to the authorities. The response of ‘do nothing’ became more pronounced, particularly for the Minangkabau and Melayu, when a village member or family member who had committed adultery was killed by a tiger (#8–11), with the Kerincinese again being split between doing nothing and reporting to the authorities.

**Table 4:** Percentage of interviewees from each ethnic group giving difference responses to each of the human-tiger conflict scenarios.

Question	Ethnicity (# responses)	Do nothing(%)	Kill tiger (%)	Report authority (%)	Relocate to another forest (%)	Permanently remove from the wild (%)	Other (%)
1 If a tiger enters your village but does nothing	Minangkabau (48)	91.7	0.0	4.2	0.0	4.2	0.0
	Kerincinese (30)	80.0	0.0	13.3	0.0	0.0	6.7
	Melayu (45)	95.6	0.0	4.4	0.0	0.0	0.0
	Other (23)	85.7	0.0	9.5	0.0	0.0	4.8
2 If livestock belonging to someone else in your village are killed by a tiger	Minangkabau (45)	20.0	46.7	11.1	6.7	13.3	2.2
	Kerincinese (26)	26.9	11.5	38.5	3.8	11.5	7.7
	Melayu (45)	46.7	31.1	20.0	2.2	0.0	0.0
	Other (24)	33.3	23.8	38.1	0.0	0.0	4.8
3 If your livestock is killed by a tiger	Minangkabau (38)	18.4	47.4	15.8	2.6	10.5	5.3
	Kerincinese (11)	36.4	9.1	36.4	0.0	18.2	0.0
	Melayu (32)	43.8	28.1	28.1	0.0	0.0	0.0
	Other (16)	25.0	25.0	43.8	0.0	0.0	6.3
4 If a man is killed by a tiger for no reason	Minangkabau (43)	20.9	46.5	14.0	2.3	14.0	2.3
	Kerincinese (30)	40.0	10.0	30.0	6.7	3.3	10.0
	Melayu (45)	44.4	35.6	20.0	0.0	0.0	0.0
	Other (22)	19.0	33.3	42.9	0.0	0.0	4.8
5 If your brother was killed by a tiger for no reason	Minangkabau (33)	33.3	51.5	6.1	3.0	6.1	0.0
	Kerincinese (11)	27.3	18.2	36.4	0.0	9.1	9.1
	Melayu (31)	41.9	35.5	22.6	0.0	0.0	0.0
	Other (15)	20.0	20.0	53.3	0.0	0.0	6.7
6 If a man was killed by a tiger while hunting (not a tiger) in the forest	Minangkabau (46)	45.7	21.7	13.0	2.2	17.4	0.0
	Kerincinese (28)	53.6	7.1	35.7	3.6	0.0	0.0
	Melayu (44)	70.5	11.4	15.9	2.3	0.0	0.0
	Other (24)	42.9	19.0	33.3	0.0	0.0	4.8
7 If your brother was killed by a tiger while hunting (not a tiger) in the forest	Minangkabau (34)	47.1	35.3	2.9	2.9	11.8	0.0
	Kerincinese (11)	36.4	9.1	45.5	0.0	9.1	0.0
	Melayu (32)	56.3	21.9	21.9	0.0	0.0	0.0
	Other (15)	33.3	13.3	46.7	0.0	6.7	0.0
8 If a woman cheated on her husband and is consequently killed by a tiger	Minangkabau (46)	71.7	13.0	8.7	2.2	2.2	2.2
	Kerincinese (29)	55.2	6.9	31.0	3.4	0.0	3.4
	Melayu (44)	72.7	9.1	15.9	0.0	2.3	0.0
	Other (24)	66.7	0.0	29.2	0.0	4.2	0.0
9 If your sister cheated on her husband and was consequently killed by a tiger	Minangkabau (34)	73.5	17.6	5.9	0.0	2.9	0.0
	Kerincinese (11)	27.3	18.2	36.4	0.0	9.1	9.1
	Melayu (32)	68.8	12.5	18.8	0.0	0.0	0.0
	Other (15)	60.0	0.0	40.0	0.0	0.0	0.0
10 If a man cheated on his wife and is consequently killed by a tiger	Minangkabau (37)	67.6	16.2	10.8	0.0	5.4	0.0
	Kerincinese (11)	36.4	18.2	36.4	0.0	0.0	9.1
	Melayu (33)	66.7	12.1	21.2	0.0	0.0	0.0
	Other (15)	66.7	0.0	33.3	0.0	0.0	0.0
11 If your brother cheated on his wife and was consequently killed by a tiger	Minangkabau (35)	65.7	20.0	5.7	2.9	2.9	2.9
	Kerincinese (11)	27.3	18.2	36.4	0.0	9.1	9.1
	Melayu (32)	65.6	12.5	21.9	0.0	0.0	0.0
	Other (15)	60.0	0.0	40.0	0.0	0.0	0.0

### Different tiger types

**Fig 1:**
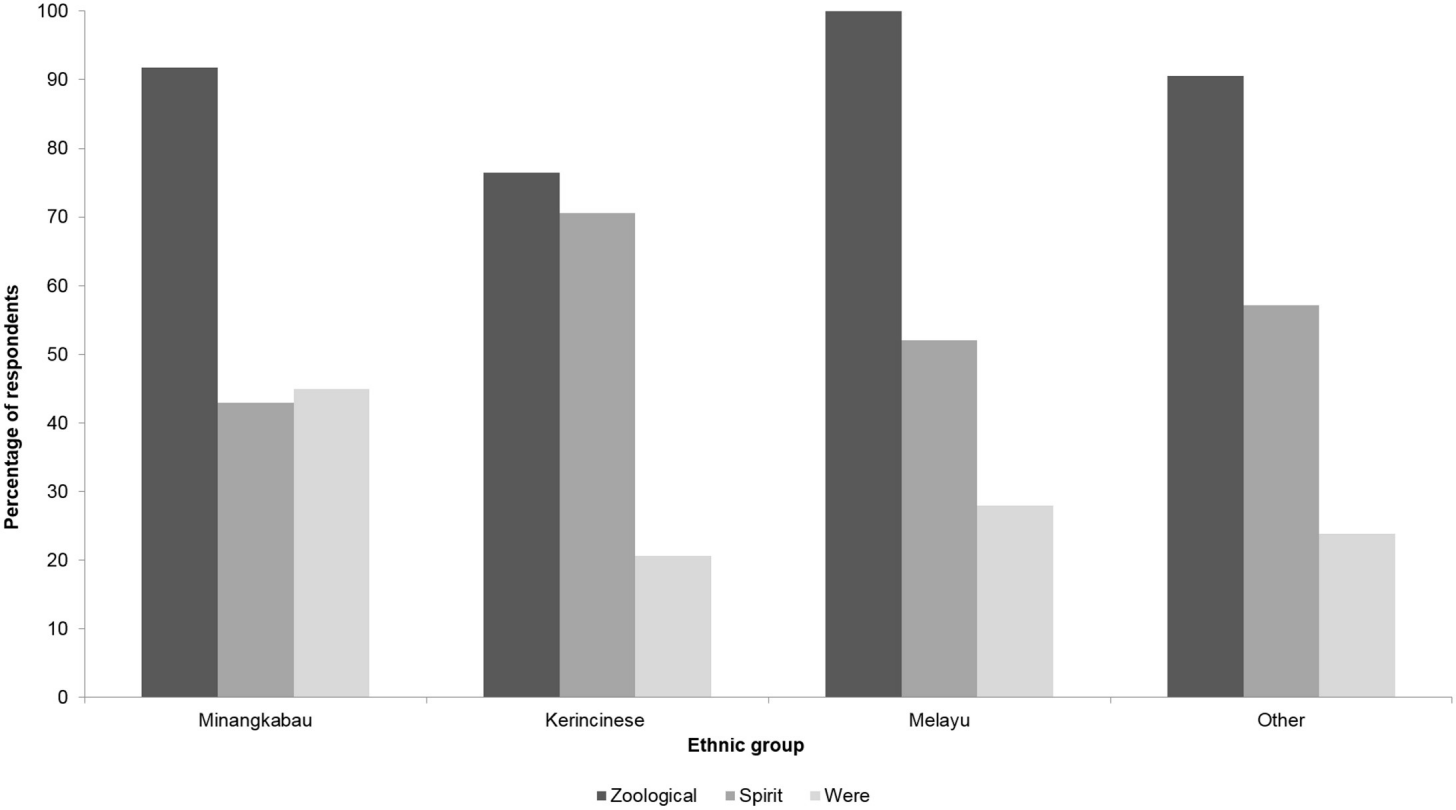
Percentage of respondents, split by ethnicity, reporting beliefs associated with zoological, spirit and weretigers (*n* = 154).

Three main types of tiger were mentioned by respondents. Most believed in the existence of a zoological tiger (90.9%) and spirit tiger (53.9%), whilst some (31.2%) mentioned the existence of weretiger (Fig 1). In describing these different types of tiger, several distinctive and reoccurring adjectives or phrases were identified. A weretiger would typically be the reincarnation of a person who had committed bad deeds in their former life and then begged God to allow them to return to earth to avoid further torture and punishment in the afterlife. Weretiger behaviour ranged from providing protection to its ancestors or their property, killing village livestock, or killing people who had a disease locally referred to as *darah buruk* (bad blood). Weretiger activities were expressed such: *“A man passed away*, *after 45 days he transformed into a weretiger… because during his life*, *he did bad things*. *This brought God’s wrath upon him*, *but it was too painful*, *so the person asked God to return to the world again and God returned him as a weretiger*. *When transformed as a tiger*, *this person eats people’s poultry and scares the villagers”*; and, *“The weretiger doesn’t disturb people*. *It takes care of his/her descendants*. *For example*, *it takes care of their grandchildren’s farm from wild boar or other pests”*.

The term “spirit tiger” was found to encompass three core elements: embodiments of ancestors; protectors of the village; and enforcers of moral codes. For example, spirit tigers may embody the soul of a villager’s ancestor, help people and guard the village: *“They* [the spirit tigers] *were the villager’s ancestors*. *They passed away then transformed to become tigers*. *Based on the elders’* [stories], *one day the tigers came to their grandchildren’s dream and told them that they transformed to be tigers and will guard the village*. *…they will show the villagers the way home when they get lost in the forest*, *or they will guard us when there is a fight between villages*.*”* Acting as an enforcer of moral rule, spirit tigers alert villagers to transgressions *“If there is someone having sex without being married*, *villagers believe that a spirit tiger will leave a* [pug] *mark in the village”*. The role of the spirit tiger as village protector and enforcer of moral rule can be combined *“There is a tiger that protects this village*. *If something bad comes to this village*, *like a disaster*, *crime or having sex without being married*, *it* [the spirit tiger] *will let the villagers know by roaring or show itself near forest edge*.*”*

## Discussion

The tiger is an apex predator, capable of killing livestock and people. However, it also embodies a wide range of spiritual attributes that transcend its animal form [25]. These spiritual transformations enable it to provide a sense of justice, order and indeed an ancestral connection, which may afford it an intangible level of protection. Supported by oral traditions (*tambo*) passed down through generations these beliefs endure to some degree amongst forest-edge communities in the Kerinci Seblat landscape who have co-existed with this large-bodied carnivore for centuries [26]. We noted that rarely was the tiger in any of its manifestations (including zoological) perceived completely negatively. For example, across all conflict scenarios, the majority response from Kerincinese and Melayu was to leave the tiger alone, rather than kill it. Such findings provide an entry point into resolving problem tiger incidents in a pro-conservation manner that increases the potential of maintaining tigers in the wild. Our findings also suggest the potential to identify ‘tiger champions’ from forest-edge communities who could help to promote tiger conservation and local-based initiatives [27].

From presenting different human-tiger conflict scenarios, with varying levels of severity towards livestock and/or human life, an unprovoked tiger attack in the village elicited the most calls for the tiger to be killed. This may be due to fear that the tiger would return and attack livestock (financial incentive) or another person (social incentive). This constrained tolerance for human loss to tiger attacks emphasises the need for vigilance, such as through community-based reporting, so that a swift response by the authorities to human-tiger encounters occur, thereby preventing further escalation [28].

From the Kerinci Seblat landscape, we found that if a village or family member was killed by a tiger whilst out hunting in the forest, the majority of respondents across all ethnic groups typically said to ‘do nothing’. This response increased further if a tiger killed someone in the village who had committed adultery or had sexual relations before marriage, suggesting that people, not the tiger, were in the wrong as they had violated a ‘pact’ between the community and the tiger by transgressing moral code. This perspective of the tiger as a punitive figure reflects the work of others who reported that at some settlements within the Kerinci Seblat landscape, tigers are located within a cosmological framework [17]. The belief in spirit tigers held by our interviewees suggests a close relationship between some people in our study area and this animal, or the ancestor/s that it is thought to embody. Our findings concur with studies that found stronger cultural beliefs for lion conservation resulted in greater tolerance towards this large carnivore in Ethiopia [29] and Kenya [30]. However, where such beliefs are not strongly held, or where fear of a large carnivore prevails, motivations for retaliatory killings may increase, as found for jaguars in Brazil [31]. It is therefore important to understand these types of social dynamics in communities living in close proximity to such species [32].

In Kerinci Seblat, the forest has long been viewed as the tiger’s domain and humans were not viewed as tiger prey [11]. Indeed, many of our interviewees said that the tiger did not seek interactions with people, although it may help them. For example, there were stories of tigers not being able to hunt for three months if they looked upon humans *“[The] tiger is a polite animal*, *it will never want to meet us face-to-face*. *Tiger is also shy*. *If a tiger accidentally meets us*, *usually it will go [away]*. *This is because if a tiger meets us*, *it will have bad luck for 40 days*. *Tiger will suffer because it can’t catch prey*.*”* Stories with common themes emerged involving tigers leading people out of the forest indirectly, such as leaving a trail of pugmarks or broken twigs and roaring whilst hidden at the entrance to the farmland.

The types of beliefs we recorded in the Kerinci Seblat landscape were also recorded in historical accounts of the colonial Dutch in the 19th century. During this period, rewards were offered to those who killed a tiger [33]. However, very few carcases were presented for rewards. This phenomenon resulted in the later discovery that the tiger was considered to be an ancestral figure and a moral force for those who violated customary law; the prevailing indigenous relationship and belief system offered tigers protection. There are many examples from Indonesia and elsewhere of social norms and taboos, enforced by informal institutions, affording wildlife protection [34]. For example, on Tinjil Island, Indonesia, taboos deter collection of water monitor lizard (*Varanus salvator*) and the reticulated python (*Python reticulatus*) targeted elsewhere for the leather market [35]; and in Madagascar taboos protect lemurs in the Indiridae family as these animals are thought to embody dead ancestors [36].

Whilst the majority of interviewees believed in the existence of tiger shaman, contrary to expectations, we did not identify any commonly cited customary laws specific to the tiger [37]. Further, we did not identify any locally perceived Islamic teachings specific to tigers and their protection, other than it falls under the wider *Khalfia* principle of human stewardship over all natural things created by God [16]. Given our targeted sampling [38] approach which deliberately sought out individuals with stronger tiger-related beliefs, our findings suggest that the strength of locally held beliefs which may offer tigers protection, may be weaker than anticipated from the anthropological literature, and highly segmented. For example, we found that customary and Islamic beliefs concerning tigers were most prevalent amongst Minangkabau, yet this did not translate to Minangkabau being more tolerant of tigers as reflected in their responses to conflict scenarios. Indeed, our scenario data suggests that Kerincinese and Melayu are more accepting of tigers. However, the results of St. John et al., (38) which reflect a broader segment of the population surrounding Kerinci Seblat National Park (systematic sample rather than targeted and snowball sampling [38]), showed no relationship between ethnicity and behavioural intention to hunt three of their four study species (wild boar, sambar deer, tiger and pangolin). Other studies have also found variations in the influence of customary laws and religion in managing real or perceived problem carnivore species, such as lion and snow leopard [39–41].

The prevailing regulation on human-wildlife conflict mitigation (including tiger) is Ministry of Forestry Regulation No. P.48/Menhut-II/2008, which was developed in a top-down manner. This regulation could be locally adapted for handling tiger incidents in the Kerinci Seblat landscape by holding stakeholder group discussions to identify locally-perceived solutions which, our findings suggest, may include maintaining tigers within the landscape rather than removing them. Furthermore, a recent study from this landscape found that pre-emptive intervention based on socio-ecological predictions could have prevented up to 51% of attacks on livestock and people and saved 15 tigers [42]. For a Critically Endangered subspecies, keeping as many tigers as possible in the Kerinci Seblat breeding population is a conservation priority [19].

## Conclusion

Local perceptions and belief systems relating to large carnivores are an important consideration for conservation practitioners and policy makers, especially where existing values may be providing some form of conservation benefit. Our study provides insights into beliefs held by a targeted section of the Kerinci Seblat community in the present day. While beliefs associated with spiritual tigers and tiger shamans remain, it is likely that such values are less prevalent than they were a century ago. Our study lends support to the importance of understanding the cultural landscape when designing conflict mitigation strategies [43] and offers support to the case for community-based conservation.

## Supporting information

S1 File Interview transcript in English. (DOCX)

S2 File Interview transcript in Indonesia. (DOCX)
