# Supplementary material for: Tolerating tigers: Gaining local and spiritual perspectives on human-tiger interactions in Sumatra through rural community interviews
Source: PLoS One. 2018 Nov 14;13(11):e0201447. doi: 10.1371/journal.pone.0201447 (PMC6235252; doi:10.1371/journal.pone.0201447)
Supplement: S1 File — (DOCX) [file pone.0201447.s001.docx]

| id number |  |
| --- | --- |

**INTERVIEW**

**Date: ­­­­­­­­­­­­____________________________________**

**Interviewer: ­­­­­­­­­­­­­­­­­­­­­­____________________________________**

**Village: ____________________________________**

**REspondent selected through** (Circle) Snowball Sampling or Questionnaire

***READ: Hello, a*s part of a research project for the University of Kent we are interested in gaining an understanding of people’s relationships with wildlife. *Anything that you tell me will only be used for the purpose of this research project and will be kept private and confidential. We will never associate your name with anything that you tell us. Are you happy to complete an interview with me?***

***Would you mind if I record the interview, as I don’t want to miss any part of our conversation and I cannot write fast enough.***

| ***Interviewee agreed to participate (tick if YES)*** |  |
| --- | --- |
| ***Interviewee agree to being recorded (tick if YES)*** |  |
| ***Recorder number*** |  |

**NUMBER OF PEOPLE PRESENT AT START OF INTERVIEW (EXCLUDING BABIES): _____________**

**NUMBER OF PEOPLE PRESENT AT end OF INTERVIEW (EXCLUDING BABIES): _____________**

| 1. | **What are your favourite animals that live in the forest?** Why do you like them? | |
| --- | --- | --- |
| 2. | **What are your least favourite animals that live in the forest?** *Why do you dislike them?* | |
|  | *Next say*: **My favourite animal is the** *[insert your favourite animal]* **and I also like the tiger and for the tiger, I am interested in hearing about what different types exist around here**. | |
| 3. | **What different types of tiger do you think live in the forest and around here where you live?**  *Write down all of the answers. If they mention zoological tiger, ask what it looks like (this is to check they are talking about tiger, not leopard). If they are confused say: “For example some people believe that the white tiger is really a ghost and has special powers”.* *If still no response move on to the next question.* | |
| 4. | **What are the characteristics of each?** *(Make sure to ask for each tiger type mentioned above).* | |
| 5. | **What happens in your village if this type of tiger is trapped or killed?** *(Ask for each tiger type mentioned above).* | |
| 6. | **Are there any stories (past/present) from your culture regarding the tiger** (*family legend etc*)? *Circle:* Yes / No ***(If NO skip to Q7).*** | |
| 6a. | | *If YES:* **How were the stories learned – from who** (*e.g. friend, relative, neighbor, rumour etc*). **When? Where?** *Get an address even if in another village.* |
| 6b. | | **Do you believe these stories?** Circle: Yes / No / Not sure |
| 6c. | | **Why do you believe/not believe these stories?** |
| 7. | **Has there been a recent tiger incident?** *(Incident means sighting, capture, killing of tiger or livestock or person. Ask for full details).* | |
| 7a. | **If all zoological tiger become extinct (die), what happens to spiritual tiger?** | |
| 8. | **Who are the most influential or most important people in your village?** *(Encourage them to list as many as possible, not just one).* | |
| 9. | **Is there a customary law and/or local belief in your village or other communities near here pertaining to the tiger?** *Circle:* Yes / No ***(If NO skip to 9f)*** | |
| 9a. | | *If YES*: **please explain** *(where, examples, ask what would happen if someone broke the customary law etc.)* |
| 9b. | | **Is this belief still followed? Could you give me an example?** *(Get dates/year & full details)* |
| 9c. | | **Do you think it is important to follow these rules today?** *If YES:* **Why?** |
| 9d. | | **Comparing today to when you were young, do you think that more, less or the same number of people in your village believe in this (*tiger*) adat that you just told me about? Why?** |
| 9e. | | **Are other rules replacing them or do you think other rules should replace them?** *(Ask them to explain their answer in detail (what, when, why, for whom etc.).* Was there a customary law or local belief about tigers in the past? *(Record details).* |
| 9f. | | **Was there a customary law or local belief about the tiger in the past?** *(Ask for details).* |
| 10. | **Is there a customary law and/or local belief in your village or other communities near here pertaining to the forest or other animal?** *Circle:* Yes / No ***(If NO skip to9f)*** | |
| 10a. | | *If YES*: **please explain** *(where, examples, ask what would happen if someone broke the customary law etc.)* |
| 10b. | | **Is this belief still followed? Could you give me an example?** *(Get dates/year & full details)* |
| 10c. | | **Do you think it is important to follow these rules today?** *If YES:* **Why?** |
| 10d. | | **Comparing today to when you were young, do you think that more, less or the same number of people in your village believe in this adat that you just told me about? Why?** |
| 10e. | | **Are other rules replacing them or do you think other rules should replace them?** *(Ask them to explain their answer in detail (what, when, why, for whom etc.)* |
| 10f. | | **Was there a customary law or local belief about the forest in the past?** *(Ask for details).* |
| 11. | **Do you know of any stories (past, present) about tigers from other ethnic Indonesian groups: Minang, Kerincense, Sundanese, Javanese, Batak, Rejang, Melayu** (C*ircle all that apply*) | |
| 11a. | | **Please could you tell me that story?** |
| 11b. | | **Do you believe these stories?** *Circle*: Yes / No |
| 11c. | | **Please could you explain why you believe/do not believe these stories?** |
| 12. | **What do you think Western people think about the tiger?** *(skip to Q13 if respondent does have anything to say here).* | |
| 12a. | | **Why do you think that they believe this?** |
| 12b. | | **Do you agree or disagree with what Western people think?**  *(circle)*: Agree / Disagree / Neutral  **Please could you explain why you feel this way?** |
| 12c. | | **Does** (*repeat what they told you above about tigers*) **offer any benefits to you?** |
| 12d. | | *If YES:* **What are these benefits?** *(Keep prompting to make sure you have included all perceived benefits).* |
| 13. | **Are there Islamic** (*or if not Muslim ask for other religion*) **beliefs or rules about the tiger?**  *Circle:* Yes / No *(If no skip to Q14)* | |
| 13a. | | *If YES:* **Can you please give me some examples** (*record ALL examples*) **of these teachings?** |
| 14. | **Can you tell me something about what the term humans as a Khalifah for the earth means to you?** | |
| 14a. | | **Do you think the tiger should be included in the concept of Khalifa?** *(If YES, ask them to elaborate).* |
| 15. | | **Do you believe in tiger shamans?** *Circle:* Yes / No |
| 15a. | | **Do you have a story or an experience that you can tell me about this?** *(record all details of experiences or stories)* |
| 16. | | **Does your village have a tiger shaman?** *Circle:* Yes / No / Don’t know **(*If NO or DON’T KNOW, skip to Q18)*** |
| 16a. | | *If YES*: **What is his name?** *Write name* |
| 16b. | | Where can he be found? *Write address* |
| 17. | | **Do you know if there ever was a tiger shaman around here? *If yes,* What was his name, how long ago was this and do you know where he lived? Will there be another one?** |
| 18. | | **Do you know if there is a tiger shaman somewhere else in Sumatra?**  *Circle:* Yes / No / Don’t know |
| 18a. | | **If yes, what is his name?** *Write name* |
| 18b. | | **Where can he be found?** *Write address* |
| 19. | **I’m now going to read you some scenarios. There are no right or wrong answers, I’m just interested in hearing what you think.** | |
| 19a. | | **A tiger is seen in the area (village, farm, forest edge, forest) but has not done anything. What do you think should happen to the tiger? Why?** |
| 19b. | | **Two men are in the forest intent on hunting illegally. They come across a tigress with her cubs. In their defense she kills one man and the other man escapes unharmed. What do you think should happen to the tiger? Why?** |
| 19c. | | **A woman who has cheated on her husband and is down by the river near the forest edge washing her clothes. A tiger comes across her and kills her. What do you think should happen to the tiger? Why?** |
| 19d. | | **A man is working on his farm and a tiger kills him for no reason.** **What do you think should happen to the tiger? Why?** |
| 19e. | | **A tiger has killed someone’s livestock. What do you think should happen to the tiger? Why?** |
| 19f. | | **A man who has cheated on his wife and is having a cigarette break after a long day work in forest edge area. A tiger comes across him and kills him. What do you think should happen to the tiger? Why?** |
| 19g. | | **Two men are in the forest intent on hunting illegally one of them is your relative. They come across a tigress with her cubs. In their defense she kills one man and the other man escapes unharmed. What do you think should happen to the tiger? Why?** |
| 19h. | | **Your relative which is woman has cheated on her husband and is down by the river near the forest edge washing her clothes. A tiger comes across her and kills her. What do you think should happen to the tiger? Why?** |
| 19i. | | **Your brother is working on his farm and a tiger kills him for no reason.** **What do you think should happen to the tiger? Why?** |
| 19j. | | **A tiger has killed your livestock. What do you think should happen to the tiger? Why?** |
| 19k. | | **Your relative which is man has cheated on his wife and is having a cigarette break after a long day work in forest edge area. A tiger comes across him and kills him. What do you think should happen to the tiger? Why?** |

| **Please complete the following questions** | | | |
| --- | --- | --- | --- |
| 106 | Gender of respondent: *(do not ask this question)* | | Male Female *(circle one)* |
| 107 | What year were you born? *(write year in full e.g. 1974)* | |  |
| 108 | What is your current occupation? | |  |
| 109 | Do you have, or do you work farm land around here? | | Yes No *(circle one)* |
| 110 | What is your level of education? *(Circle one)* | | No school  Elementary  Junior high  Senior high  University |
| 111a | Are you Muslim? | | Yes No *(circle one)* ***(If YES, skip to Q112a)*** |
| 111b | | What is your religion? |  |
| 112a | How do you describe your ethnic origin e.g. are you Sundanese, Javanese, Kerincenese, Minangkabau or mixed? | | ***(If MIXED ask Q112b,c, otherwise skip to Q113a)*** |
|  | | How do you describe the ethnic origin of your parents e.g. Sundanese, Javanese, Kerincenese, Minangkabau or mixed? | **112b)** Father:  **112c)** Mother:  *(If either parent MIXED please specify)* |
| 113a | Were you born in this village? *(circle one option)* | | Yes No **(*if YES, skip to Q114)*** |
| 113b | | Which village were you born in? |  |
| 113c | | How long have you lived in this village? |  |

End: Thank you very much for your time. Having completed this interview and understanding the kind of stories I would like to hear, do you know of anyone else I may speak with? ( *Write down name, address, hp number etc.*)­­­­­­
