# Supplementary material for: Tolerating tigers: Gaining local and spiritual perspectives on human-tiger interactions in Sumatra through rural community interviews
Source: PLoS One. 2018 Nov 14;13(11):e0201447. doi: 10.1371/journal.pone.0201447 (PMC6235252; doi:10.1371/journal.pone.0201447)
Supplement: S2 File — (DOCX) [file pone.0201447.s002.docx]

| id number |  |
| --- | --- |

**INTERVIEW**

**tanggal: ­­­­­­­­­­­­____________________________________**

**pewawancara: ­­­­­­­­­­­­­­­­­­­­­­ ____________________________________**

**desa: ____________________________________**

**REspondent dipilih melalui (Circle) Snowball Sampling atau Questionnaire**

***READ: Salam, sebagai bagian University of Kent di UK (Inggris), kami sedang mengetahui melakukan penelitian untuk mengetahui bagaimana hubungan manusia dan satwa liar.***

***READ: Semua jawaban yang akan bapak/ibu berikan kepada saya hanya akan digunakan untuk penelitian ini dan akan tetap dijaga kerahasiaannya. Kami tidak akan mengaitkan bapak/ibu dengan apapun atas jawaban yang bapak/ibu berikan kepada kami.***

***Apakah saya boleh merekam interview ini? Ini karena saya tidak ingin terlewatkan sedikitpun dari perbincangan kita dan saya tidak dapat menulis dengan cepat.***

| ***Apakah bapak mau mengikuti survey ini? (tick jika Ya)*** |  |
| --- | --- |
| ***Apakah tidak apa-apa jika saya merekanya? (tick jika Ya)*** |  |
| ***Nomer interview*** |  |

**jumlah masyarakat yang hadir saat interview dimulai (diluar dari bayi): _____________**

**jumlah masyarakat yang hadir saat intervew selesai (diluar dari bayi): _____________**

| 1. | **Hewan apakah yang anda suka yang hidup di hutan?** Kenapa anda suka dengan hewan tersebut***?*** | |
| --- | --- | --- |
| 2. | **Hewan apakah yang paling tidak anda sukai di hutan?** *kenapa anda tidak menyukainya?* | |
|  | *Next say*: **Hewan kesukaan saya adalah** *[Masukkan hewan kesukaan kamu]* dan saya juga suka harimau. Dan untuk harimau, saya tertarik untuk mendengar tentang jenis apa saja yang ada di sekitar sini | |
| 3. | **Harimau jenis apa saja yang hidup di hutan dan disekitar tempat anda tinggal?**  *Tuliskan semua jawaban. Jika mereka menjawab Harimau (dalam arti sesungguhnya),tanyakan seperi apa penampakanya (ini untuk mengecheck bahwa mereka bicara tentang harimau bukan macan dahan).* *Jika mereka bingung jelaskan. “Contohnya, beberapa orang percaya bahwa harimau putih adalah hantu dan memiliki kekuatan tertentu. Jika masih tidak mendapatkan jawaban, lanjutkan ke pertanyaan selanjutnya.* | |
| 4. | **Apa ciri-ciri dari masing-masing dari harimau tersebut?** *(Pastikan bahwa setiap jenis harimau yang disebutkan diatas dijelaskan ciri-cirinya)* | |
| 5. | **Apa yang terjadi di desa anda jika jenis harimau ini terjerat atau terbunuh di desa ini?** *(tanyakan untuk setiap harimau yang disebutkan di atas (no 3)* | |
| 6. | **Apakah ada cerita (jaman dulu/sekarang) dari suku/budaya anda terkait dengan harimau** *(cerita lama dari keluarga, dll)?* **Lingkari: Ya Tidak (Jika tidak skip ke Q7)** | |
| 6a. | | *Jika ya,* **bagaimana anda mengetahui ceritanya** *–* **dari siapa** *(cth: teman, keluarga, tetangga, isu, dll).* **Kapan? Dimana?** *(Tanyakan alamatnya meskipun orang tersebut di desa lain* |
| 6b. | | **Apakah anda percaya dengan cerita tersebut? Lingkari:** Ya Tidak tidak yakin |
| 6c. | | **Kenapa percaya? Kenapa tidak percaya?** |
| 7 | **Apakah baru-baru ini ada kejadian terkait dengan harimau** *(kejadian: harimau terlihat, tertangkap, terbunuh, ternak terbunuh atau orang terbunuh. Tanyakan detail)* | |
| 7a | **Jika harimau (zoological tiger) punah (mati semua) apa yang akan terjadi pada harimau jadi-jadian (spiritual tiger)?** | |
| 8. | **Siapakah orang-orang yang paling berpengaruh atau paling penting di kampung anda? *(****Minta mereka menyebutkan sebanyak mungkin, tidak hanya satu)* | |
| 9. | **Apakah ada adat dan atau kepercayaan setempat di kampung anda atau masyarakat dekat sini terkait dengan harimau?** Lingkari: Ya Tidak. **(Jika tidak lanjut ke-9e)** | |
| 9a. | | *Jika Ya*: **tolong jelaskan** *(dimana,contoh,tanyakan jika seseorang melanggar hukum adat etc.)* |
| 9b. | | **Apakah kepercayaan/hukum adat ini masih diikuti? Dapatkah anda memberikan contoh?** *(tanyakan tanggal/tahun dan &keterangan detil)* |
| 9c. | | **Menurut anda, sekarang ini apakah penting untuk mengikuti aturan dalam kepercayaan tersebut?** *Jika ya/tidak***, kenapa?** |
| 9d. | | **Dibandingkan ketika anda masih muda, menurut anda apakah saat ini lebih banyak, kurang, atau sama jumlahnya, orang di kampung anda yang percaya dengan kepercayaan (harimau) yang baru saja anda ceritakan pada saya?** |
| 9e. | | **Apakah ada aturan lain yang menggantikannya atau adakah aturan lain yang seharusnya menggantikannya?** *Minta mereka untuk menjelaskan jawaban mereka sedetail mungkin (apa, kapan, untuk siapa, dsb).* |
| 9f. | | Apakah dulu ada hukum adat atau kepercayaan tentang harimau (tanyakan detail) |
| 10. | **Apakah ada kepercayaan local yang pernah ada di kampung anda yang terkait dengan hewan lainnya atau dengan hutan?** Lingkari: Ya Tidak. **(Jika tidak, lanjut ke 10e)** | |
| 10a. | | *Jika Ya:* ***tolong jelaskan*** *(dimana,contoh,tanyakan jika seseorang melanggar hokum adat etc.)* |
| 10b. | | **Apakah kepercayaan/hukum adat ini masih diikuti? Dapatkah anda memberikan contoh?** *(tanyakan tanggal/tahun dan &keterangan detil)* |
| 10c. | | **Menurut anda, sekarang ini apakah penting untuk mengikuti aturan dalam kepercayaan tersebut?** *Jika ya*, **kenapa?** |
| 10d. | | **Dibandingkan ketika anda masih muda, menurut anda apakah saat ini lebih banyak, kurang, atau sama jumlahnya, orang di kampung anda yang percaya dengan aturan/kepercayaan lokal yang baru saja anda ceritakan pada saya?** |
| 10e. | | **Apakah ada aturan lain yang menggantikannya atau adakah aturan lain yang seharusnya menggantikannya?** *Minta mereka untuk menjelaskan jawaban mereka sedetail mungkin (apa, kapan, untuk siapa, dsb).* Apakah ada hukum adat atau kepercayaan tentang harimau di masa lalu? *(Record details).* |
| 10f. | | Apakah dulu ada hukum adat atau kepercayaan tentang hewan lain atau hutan (tanyakan detail) |
| 11. | **Apakah anda mengetahui cerita apapun tentang harimau (dulu dan sekarang) dari suku lain di Indonesia : Minang, Krinci, Sunda, Jawa, Batak, Rejang, melayu (lingkari jika mereka tau)** | |
| 11a. | | **Dapatkah anda menceritakanya?** |
| 11b. | | **Apakah anda percaya cerita tersebut? Lingkari : Ya Tidak** |
| 11c. | | **Kenapa anda percaya?/kenapa tidak percaya?** |
| 12. | **Menurut pengetahuan anda apa yang bule pikirkan tentang harimau** | |
| 12a. | | **Menurut anda mengapa mereka percaya hal tersebut?** |
| 12b. | | **Apakah anda setuju atau tidak setuju dengan yang dipikirkan *bule*?**  *(lingkari)*: setuju / tidak setuju / Neutral  **Bisakah anda jelaskan kenapa anda merasa seperti ini?** |
| 12c. | | **Apakah menurut anda kepercayaan tersebut** (ulangi apa yang mereka ucapkan diatas) **memberikan manfaat buat anda** |
| 12d. | | *Jika Ya,* **apa saja manfaatnya?** *(Terus dorong mereka untuk menyebutkan semua manfaatnya dan pastikan Anda telah mencantumkan semua manfaat yang dirasakan)* |
| 13. | **Apakah ada kepercayaan atau peraturan dalam agama islam** *(atau agama lain-jika orangnya bukan muslim)* **tentang harimau?** *Lingkari*: Ya Tidak ***Jika tidak lanjutkan ke pertanyaan Q14*** | |
| 13a. | | *Jika ya,* **tanyakan:bisakah anda memberikan contoh dari ajaran ini?** *(pastikan untuk* ***merekam*** *SEMUA contoh)* |
| 14. | **Bisakah anda menceritakan kepada saya bagaimana anda memaknai konsep “manusia adalah khalifah dimuka bumi”** | |
| 14a. | | **Menurut anda apakah harimau merupakan bagian di dalamnya?** *(If Ya,tanyakan detil).* |
| 15. | | **Apakah anda percaya kepada dukun/pawang harimau?** Lingkari: Ya atau Tidak |
| 15a. | | **Apakah anda memiliki cerita atau pengalaman yang dapat anda ceritakan kepada saya mengenai hal ini?** Rekam keterangan detil |
| 16. | | **Apakah di kampung anda memiliki dukun/pawang harimau?** Lingkari: Ya atau Tidak ***(Jika tidak, lanjutkan ke Q18)*** |
| 16a. | | *IJika ya,* **siapa namanya***? tuliskan namanya ______* |
| 16b. | | Dimana dia bisa ditemukan? Tuliskan alamatnya________ |
| 17. | | **Apakah anda mengetahui jika dulu pernah ada dukun/pawang harimau (yang telah meninggal) disekitar sini? Jika iya, siapa namanya? Berapa tahun yang lalu dan apakah anda tau dimana dulu dia tinggal? Apakah akan ada pengantinya?** |
| 18. | | **Apakah anda tau pawang/dukun harimau di tempat lain di Sumatera?lingkari :** Ya / Tidak |
| 18a. | | **Jika iya , siapa namanya?** *Tulis namanya* |
| 18b. | | **Dimana pawang itu bisa dijumpai?** *Tulis alamat (provinsi)* |
| 19. | **Setelah ini saya akanmembacakan beberapa skenario. Tidak ada jawaban yang benar atau salah. Saya hanya ingin mengetahui apa yang anda pikirkan** | |
| 19a. | | **Harimau terlihat di sekitar sini (kampung, ladang, batas hutan, hutan) tetapi tidak melakukan apapun. Menurut anda apa yang harus dilakukan terhadap harimaunya? kenapa?** |
| 19b. | | **Dua orang di dalam hutan bermaksud berburu secara illegal. Mereka bertemu seeor harimau dengan anak-anaknya. Dalam mempertahankan dirinya, harimau itu membunuh salah satu dari orang itu dan yang satunya melarikan diri dan selamat. Menurut anda apa yang harus dilakukan terhadap harimaunya? kenapa?** |
| 19c. | | **Seorang wanita yang berselingkuh (menghianati suaminya) dan pergi ke sungai yang dekat dengan tepi hutan mencuci bajunya. Seekor harimau mendatanginya dan membunuhnya. Menurut anda apa yang harus dilakukan terhadap harimaunya? kenapa?** |
| 19d. | | **Seorang laki-laki sedang bekerja di ladangnya dan seekor harimau membunuhnya tanpa alasan. Menurut anda apa yang harus dilakukan terhadap harimaunya? kenapa?** |
| 19e. | | **Seekor harimau membunuh ternak warga.** **Menurut anda apa yang harus dilakukan terhadap harimaunya? kenapa?** |
| 19f. | | **Seorang laki-laki berselingkuh, pada suatu waktu dia sedang merokok di pinggir hutan. Seekor harimau mendatanginya dan membunuhnya. Menurut anda apa yang harus dilakukan terhadap harimaunya? kenapa?** |
| 19g. | | **Dua orang di dalam hutan bermaksud berburu secara illegal salah satunya saudara bapak/ibu. Mereka bertemu seekor harimau dengan anak-anaknya. Dalam mempertahankan dirinya, harimau itu membunuh saudara anda dan yang satunya melarikan diri dan selamat. Menurut anda apa yang harus dilakukan terhadap harimaunya? kenapa?** |
| 19h. | | **Saudara perempuan anda berselingkuh (menghianati suaminya) dan pergi ke sungai yang dekat dengan tepi hutan mencuci bajunya. Seekor harimau mendatangia dan membunuhnya. Menurut anda apa yang harus dilakukan terhadap harimaunya? kenapa?** |
| 19i. | | **Saudara laki-laki anda sedang bekerja di ladangnya dan seekor harimau membunuhnya tanpa alasan. Menurut anda apa yang harus dilakukan terhadap harimaunya? kenapa?** |
| 19j. | | **Seekor harimau membunuh ternak anda.** **Menurut anda apa yang harus dilakukan terhadap harimaunya? kenapa?** |
| 19k. | | **Saudara laki-laki anda berselingkuh, pada suatu waktu dia sedang merokok di pinggir hutan. Seekor harimau mendatanginya dan membunuhnya. Menurut anda apa yang harus dilakukan terhadap harimaunya? kenapa?** |

| **Lengkapi pertanyaan berikut** | | | |
| --- | --- | --- | --- |
| 106 | Jenis kelamin responden: *(jangan tanyakan pertanyaan ini )* | | Laki-laki Perempuan *(lingkari salah satu)* |
| 107 | Tahun berapa anda lahir? *(tulis secara lengkap e.g. 1974)* | |  |
| 108 | Apa pekerjaan bapak? | |  |
| 109 | Apakah bapak punya ladang/sawah atau bekerja di ladang di sekitar sini? | | Ya Tidak *(lingkari salah satu)* |
| 110 | Pendidikan terakhir? *(lingkari salah satu)* | | NA SD SMP SMA Universitas |
| 111a | Apakah anda Muslim?? | | Ya Tidak (lingkari salah satu) (jika iya, SKIP ke ***Q112a*** |
| 111b | | Apakah agama anda? |  |
| 112a | Anda suku apa? e.g. apakah anda Sunda, Jawa, Kerinci, Minangkabau atau campuran? | | *(jika* ***campuran*** *tanyakan* ***Q112b****,****c*** *jika tidak lewatkan ke* ***Q113a****)* |
|  | Bagaimana anda menjelaskan etnis asli anda? e.g. Sunda, Jawa, Kerinci, Minangkabau atau percampuran? | | **112b)** Ayah:  **112c)** Ibu:  *(jika orang tua juga percampuran tolong dijelaskan)* |
| 113a | Apakah anda lahir di desa ini? *(lingkari salah satu)* | | Ya Tidak (jika tidak tanyakan **Q113b,c**) |
| 113b | | Dari desa mana anda lahir?  (province) |  |
| 113c | | Berapa lama anda tinggal di desa ini? |  |

**Penutup:** terima kasih banyak atas waktu anda sehingga saya bisa melakukan interview secara lengkap dan mendengarkan cerita yang ingin saya dengar. Apakah anda tahu orang lain yang kira-kira bisa saya interview juga? (tuliskan nama, alamat, dan nomor HP)
